# Long-term dental care utilization trajectories as behavioral markers of cognitive aging: a 14-year national cohort study

**DOI:** 10.3389/fpubh.2026.1938854

**Published:** 2026-09-24

**Authors:** Young-Suk Jung, Hyunjoo Joo

**Affiliations:** 1 Department of Periodontology, School of Dentistry, Chonnam National University, Gwangju, Republic of Korea; 2 Leader's Dental Clinic, Jeju, Republic of Korea; 3 Department of Preventive Medicine, College of Medicine, Dankook University, Cheonan, Republic of Korea

**Keywords:** activities of daily living, cognitive aging, cognitive decline, dental care utilization, group-based trajectory modeling, Health and Retirement Study, mediation analysis, older adults

## Abstract

**Introduction:**

Dental care utilization may serve as a behavioral marker of healthy aging, but previous studies have largely relied on single-time-point measures. Whether long-term utilization patterns are associated with cognitive aging and through which functional pathways remains unclear.

**Methods:**

We analyzed eight biennial waves (2006–2020) of the Health and Retirement Study. Group-based trajectory modeling identified dental care utilization trajectories among 14,599 adults aged ≥50 years at baseline. Cognitive function was assessed using a 27-point summary score. Linear mixed-effects models estimated associations between trajectory and cognitive decline. Serial mediation analysis using empirical Bayes individual slope estimates examined mediation through longitudinal changes in self-rated health (SRH) and activities of daily living (ADL) limitations, with Persistent High as the reference.

**Results:**

Three trajectories were identified: Persistent High (53.3%), Decreasing (14.5%), and Persistent Low (32.2%). Compared with Persistent High, both suboptimal trajectories showed faster cognitive decline (Decreasing: *β* = −0.085 per 2-year wave, 95% CI − 0.118 to −0.053; Persistent Low: *β* = −0.107, 95% CI − 0.133 to −0.081; both *p* < 0.0001), corresponding to approximately 0.60 and 0.75 additional points of decline over 14 years, respectively, against a 2.29-point decline in the Persistent High group. The difference between the Persistent Low and Decreasing trajectories was not significant (*β* difference = −0.0217, 95% CI − 0.0580 to 0.0146; *p* = 0.241). Worsening ADL limitations accounted for 9.3 and 11.6% of the associations for the Decreasing and Persistent Low trajectories, respectively. In a lagged analysis separating exposure and mediator assessment (2006–2016) from outcome assessment (2016–2020), the direct associations and ADL-mediated pathway remained significant, whereas the SRH-to-cognition pathway did not.

**Conclusion:**

Persistently low and declining dental care utilization were associated with similarly faster cognitive decline, with functional deterioration representing the principal mediating pathway. Long-term dental care utilization may be an accessible behavioral marker of broader functional vulnerability. Although modest, these associations may have public health relevance because utilization can be captured through surveys and administrative or claims data. Causal inference cannot be established from this observational study.

## 1 Introduction

Population aging has made the preservation of cognitive health a major public health priority. Alongside established vascular and metabolic risk factors, increasing attention has been directed toward modifiable behavioral markers that might help identify older adults at increased risk of cognitive decline before clinically apparent impairment develops. Oral health has emerged as one such marker, with longitudinal studies consistently showing that periodontal disease and tooth loss are associated with an increased risk of cognitive decline and dementia, although the observed associations are generally modest (1–4). Recent longitudinal studies have reinforced this link, reporting that poorer oral health and reduced chewing ability predict incident cognitive impairment and dementia (5), and that periodontal treatment is associated with slower cognitive decline (6).

Several biological mechanisms have been proposed. Chronic periodontal inflammation may promote neurodegeneration through systemic dissemination of inflammatory mediators, including interleukin-6 and C-reactive protein, both implicated in Alzheimer’s disease pathology (7). *Porphyromonas gingivalis*, a keystone periodontal pathogen, together with its toxic proteases, has been detected in the brains of individuals with Alzheimer’s disease (8). A complementary, non-inflammatory pathway involves impaired mastication, which may reduce sensory and vascular stimulation to brain regions supporting learning and memory (9). These mechanisms are likely to coexist rather than operate independently.

More broadly, the concept of oral frailty—a reversible early decline in oral function now formally defined by consensus (10)—has emerged as a marker of subsequent functional and cognitive deterioration in older adults. Longitudinal cohorts have linked oral frailty to incident mild cognitive impairment (11) and to unfavorable trajectories of cognitive function and psychological well-being (12), and oral functional limitation predicts the onset of physical frailty (13).

Despite this evidence, most epidemiological studies have relied on static clinical indicators measured at a single time point. Dental care utilization represents a broader behavioral construct, reflecting engagement with preventive health care, access to services, and the capacity to sustain health-promoting behaviors over time. Lower socioeconomic status, limited access to dental care, and depressive symptoms have all been associated with reduced dental attendance (14–16). Longitudinal patterns of dental care utilization may therefore provide insight into broader processes of healthy aging beyond oral health status alone. Importantly, dental care utilization is not static in later life: some individuals maintain regular preventive visits, whereas others experience substantial declines as their health and functional status change.

Group-based trajectory modeling provides a person-centered approach for identifying distinct longitudinal patterns of health-related behaviors rather than assuming a single average trajectory (17, 18). Although trajectory modeling has recently been applied to dental attendance in the general adult population (19) and to dental care utilization in specific subpopulations of the Health and Retirement Study (20), whether long-term dental care utilization trajectories are associated with cognitive aging, and through which functional pathways these associations may operate, remains unknown. Functional decline is a well-established predictor of cognitive deterioration (21, 22), whereas self-rated health provides a complementary indicator of overall health that independently predicts adverse outcomes (23).

Using 14 years of longitudinal data from the Health and Retirement Study, we identified distinct trajectories of dental care utilization, examined their associations with the rate of cognitive decline, and evaluated whether longitudinal changes in self-rated health and activities of daily living mediated these associations. To address the temporal ambiguity inherent in concurrently measured constructs, we additionally conducted a lagged analysis in which the outcome window was separated from the exposure and mediator windows.

## 2 Materials and methods

### 2.1 Data source and study population

Data were drawn from the Health and Retirement Study (HRS), a nationally representative biennial cohort study of community-dwelling U. S. adults aged ≥50 years (24), using the RAND HRS Longitudinal File (version 2022v1). Eight biennial waves spanning 2006–2020 (waves 8–15) were analyzed.

Of all respondents in the RAND file (*N* = 45,234), we retained those who completed the 2006 (wave 8) core interview as self-respondents and were aged ≥50 years at that time (*n* = 17,991). Because group-based trajectory modeling estimates class membership from all available repeated observations under a missing-at-random assumption, we further required dental care utilization data in at least three of the eight waves, balancing sample representativeness against the reliability of trajectory estimation. Exclusion at this step (*n* = 3,392) was driven predominantly by death and loss to follow-up over the 14-year observation period. The final analytic sample comprised 14,599 participants (Supplementary Figure S1). Characteristics of included and excluded eligible respondents are compared in Supplementary Table S2.

The HRS was approved by the Institutional Review Board of the University of Michigan, and all participants provided written informed consent. This secondary analysis used publicly available de-identified data and was exempt from additional institutional ethical approval.

### 2.2 Measures

Dental care utilization. At each wave, participants reported whether they had visited a dentist for treatment, cleaning, or a routine check-up during the previous 2 years (or since the last interview). Responses were coded as binary (yes/no).

Cognitive function. Global cognitive function was assessed at each wave using a 27-point summary score comprising immediate and delayed 10-word recall (0–20), serial 7 subtraction (0–5), and backward counting from 20 (0–2). Higher scores indicate better cognition. This 27-point summary score is a validated and widely used measure of global cognition in the Health and Retirement Study and underlies the Langa–Weir classification of cognitive status; it has been applied extensively in longitudinal studies of cognitive aging. Our aim was to characterize the rate of cognitive change at the population level rather than to establish a clinical diagnosis, for which this measure is well suited. It is administered to self-respondents at all ages and in all waves of the study period. We did not use the 35-point RAND total cognition score because its mental-status component is administered only to respondents aged ≥65 years and is unavailable in the 2018 and 2020 waves; using it would have produced structural missingness for respondents aged 50–64 years and would have required combining non-equivalent composites across waves (25). Cognitive scores were treated as missing for waves in which the interview was completed by a proxy.

Self-rated health. Self-rated health (SRH) was assessed on a five-point scale from 1 (excellent) to 5 (poor), with higher scores indicating poorer perceived health (23).

Functional limitation. Functional limitation was assessed using the RAND HRS summary count of difficulties in five activities of daily living (ADL; range 0–5), with higher scores indicating greater limitation.

Covariates. Baseline covariates in the primary models were age, sex, educational attainment, and race/ethnicity (White, Black, Other). Depressive symptoms (8-item Center for Epidemiologic Studies Depression Scale, CES-D; range 0–8) (26), baseline cognitive function, household income, and household wealth were additionally adjusted for in sensitivity analyses.

### 2.3 Statistical analysis

Trajectory identification. Group-based trajectory modeling (GBTM) was used to identify distinct longitudinal patterns of dental care utilization (17, 18). Models were fitted with the hlme function of the lcmm package in R (27), specifying class-specific intercepts and linear slopes without within-class random effects. Because dental care utilization was binary, trajectories were modeled on the probability scale using a Gaussian identity-link specification. Solutions with two to six classes were estimated using a grid of random starting values, and were compared on the Bayesian Information Criterion (BIC), entropy, average posterior probability (APP) of assignment, the presence of empty classes, and the size of the smallest class (Supplementary Table S1). Participants were assigned to the class with the highest posterior probability. Class-specific slopes were modeled as linear. A quadratic specification was additionally examined; although it yielded a modestly lower Bayesian Information Criterion (74,800 versus 75,034 for the three-class linear solution), it produced the same three trajectories with nearly identical class membership (97.5% agreement) and an equivalent ordinal interpretation (persistently high, declining, persistently low). The more parsimonious and readily interpretable linear specification was therefore retained for the primary analysis (Supplementary Table S1).

Baseline characteristics were compared using one-way analysis of variance for normally distributed continuous variables, the Kruskal–Wallis test for CES-D scores, and the chi-square test for categorical variables, including the presence of one or more ADL limitations.

Primary analysis. The association between trajectory group and cognitive change was estimated with a linear mixed-effects model including a trajectory × time interaction, random intercepts and random slopes, adjusted for baseline age, sex, education, and race/ethnicity. Time was coded 0–7 (one unit = one biennial wave = two years). All available observations were used under a missing-at-random assumption. Degrees of freedom were obtained by Satterthwaite approximation. The difference between the Persistent Low and Decreasing interaction coefficients was tested with a Wald contrast.

Mediation analysis. Separate linear mixed-effects models with random intercepts and random slopes were fitted for SRH, ADL limitation, and cognitive function, and empirical Bayes estimates of individual slopes were extracted. Serial multiple mediation analysis was then conducted with the lavaan package (28), with the Persistent High trajectory as the reference. The model specified paths from trajectory group to the SRH and ADL slopes, from the SRH slope to the ADL slope, and from both mediator slopes to the cognitive slope, adjusting for baseline age, sex, education, and race/ethnicity. Direct, single-mediator indirect, serial indirect, and total indirect effects were estimated, with 95% confidence intervals estimated from 5,000 bootstrap resamples (29), together with a Wald contrast of the two direct effects. Full-information maximum likelihood was used for missing outcome data. Because individual slopes were treated as observed variables in the structural model, first-stage estimation uncertainty was not formally propagated; the resulting standard errors should therefore be regarded as approximate and the mediation estimates as descriptive rather than definitive. Because empirical Bayes slope estimates are shrunk toward the mean, coefficients from this model are expected to be smaller in magnitude than those from the primary mixed-effects model; the two are reported separately.

Sensitivity analyses. Eleven sensitivity analyses were conducted (Supplementary Tables S3–S13): survey weight–adjusted estimation; additional adjustment for baseline cognition; further adjustment for baseline depressive symptoms; full adjustment additionally including household income and wealth; exclusion of participants with low baseline cognition; a lagged design in which trajectory membership and mediator slopes were estimated over 2006–2016 (waves 8–13) and the cognitive slope over 2016–2020 (waves 13–15); additional adjustment for baseline current smoking, any alcohol consumption, and marital/partnership status; three analyses progressively excluding participants with prevalent or incident cognitive impairment to address possible reverse causation; and an alternative specification in which the baseline ADL limitation count was modeled as a covariate rather than longitudinal change in ADL limitations as a mediator. Baseline cognitive status was classified using established 27-point cut-points (0–6 dementia, 7–11 cognitive impairment no dementia (CIND), and 12–27 normal cognition); these analyses excluded, in turn, participants with baseline dementia, CIND, or stroke; those additionally developing cognitive impairment by wave 9 (~2 years); and those additionally developing cognitive impairment through wave 10 (~4 years).

All analyses were performed in R version 4.5.2. Statistical significance was defined as a two-sided *p* < 0.05.

## 3 Results

### 3.1 Trajectories of dental care utilization

Among 14,599 respondents, group-based trajectory modeling identified three distinct trajectories of dental care utilization (Figure 1). The three-class solution was selected on the basis of overall model fit, classification quality, and interpretability (Supplementary Table S1). Although the six-class solution had a lower BIC than the three-class solution, the four-, five-, and six-class solutions each contained one or more empty classes and showed markedly degraded classification quality (entropy 0.56–0.64; minimum APP 0.36–0.49). Among solutions with no empty class and entropy above the conventional 0.80 threshold, the three-class model had the lowest BIC (75,034 vs. 81,116 for the two-class model), with entropy 0.881, a smallest class of 14.5%, and APPs ranging from 0.833 to 0.967.

**Figure 1:**
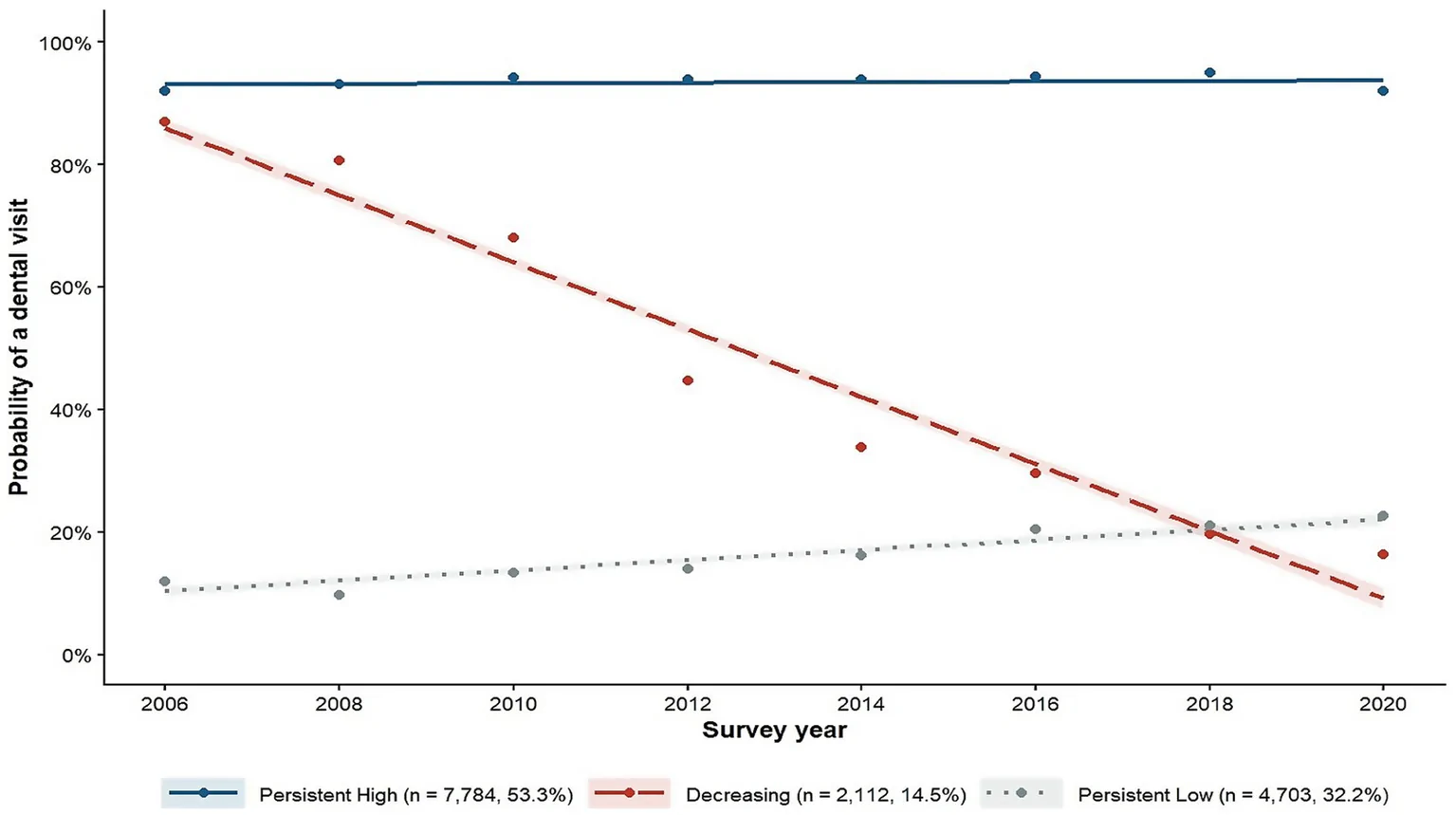
Trajectories of dental care utilization over the 2006–2020 study period. Lines represent predicted probabilities with 95% confidence intervals, and points represent observed proportions.

The three groups were labeled Persistent High (*n* = 7,784; 53.3%), Decreasing (*n* = 2,112; 14.5%), and Persistent Low (*n* = 4,703; 32.2%). The Decreasing group began with a high probability of dental attendance that fell substantially over follow-up. The Persistent Low group showed a modest upward drift in attendance over the observation period but remained far below the Persistent High group throughout; it is therefore labeled by its sustained low level rather than by the absence of any change.

### 3.2 Baseline characteristics

Baseline characteristics differed significantly across trajectory groups (Table 1). Participants in the Persistent High group had the highest educational attainment (13.60 years), the highest baseline cognitive score (16.73), and the best self-rated health (2.50). The Persistent Low group had the lowest educational attainment (10.93 years) and baseline cognition (13.80), the poorest self-rated health (3.17), and the highest proportion of Black participants (21.6% vs. 8.2%). The Decreasing group was intermediate on all baseline measures (education 12.21 years; cognition 14.94), entering follow-up with a more favorable profile than the Persistent Low group despite subsequently experiencing a comparable rate of cognitive decline.

**Table 1:** Baseline characteristics of participants by dental care utilization trajectory.

Characteristic	Overall (*n* = 14,599)	Persistent high (*n* = 7,784)	Decreasing (*n* = 2,112)	Persistent low (*n* = 4,703)	*p*
Age, years, mean (SD)	67.45 (9.64)	66.53 (9.52)	67.41 (9.66)	69.01 (9.65)	<0.001
Female, *n* (%)	8,627 (59.1)	4,712 (60.5)	1,219 (57.7)	2,696 (57.3)	0.001
Education, years, mean (SD)	12.54 (3.22)	13.60 (2.72)	12.21 (3.09)	10.93 (3.33)	<0.001
Race/ethnicity, *n* (%)					<0.001
White	10,884 (74.6)	6,501 (83.5)	1,448 (68.6)	2,935 (62.4)	
Black	2,011 (13.8)	641 (8.2)	353 (16.7)	1,017 (21.6)	
Other	1,703 (11.7)	642 (8.2)	311 (14.7)	750 (16.0)	
Depressive symptoms (CES-D, 0–8), median [IQR]	1.00 [0.00, 2.00]	0.00 [0.00, 2.00]	1.00 [0.00, 2.00]	1.00 [0.00, 3.00]	<0.001
Self-rated health (1–5), mean (SD)	2.77 (1.09)	2.50 (1.01)	2.86 (1.07)	3.17 (1.08)	<0.001
≥1 ADL limitation, *n* (%)	2,036 (13.9)	721 (9.3)	331 (15.7)	984 (20.9)	<0.001
Cognitive score (0–27), mean (SD)	15.55 (4.42)	16.73 (3.92)	14.94 (4.36)	13.80 (4.62)	<0.001

Counts may not sum to the total because of missing data for some variables, and percentages may not sum to 100 because of rounding. Continuous variables were compared using ANOVA or the Kruskal–Wallis test, and categorical variables were compared using the chi-square test. Higher self-rated health scores indicate poorer perceived health. Baseline ADL limitations are presented as the number (%) of participants with ≥1 limitation because the ADL limitation count was highly right-skewed, with a median [IQR] of 0 [0, 0] in all groups; the *p* value was obtained using the chi-square test. ADL, activities of daily living; CES-D, Center for Epidemiologic Studies Depression Scale; IQR, interquartile range; SD, standard deviation.

### 3.3 Cognitive decline according to dental care utilization trajectory

In the linear mixed-effects model, the Persistent High trajectory had a cognitive slope of −0.3272 points per 2-year wave (95% CI − 0.3419 to −0.3125), equivalent to a decline of approximately 2.29 points over the 14-year follow-up. Both other groups declined significantly faster (Decreasing: additional *β* = −0.0853, 95% CI − 0.1181 to −0.0525; Persistent Low: additional *β* = −0.1070, 95% CI − 0.1329 to −0.0811; both *p* < 0.0001), corresponding to an additional 0.60 and 0.75 points of decline, respectively (Table 2, Figure 2). A formal contrast showed no statistically significant difference between the two trajectory coefficients (*β* difference = −0.0217, 95% CI − 0.0580 to 0.0146, *p* = 0.241).

**Table 2:** Cognitive decline by dental care utilization trajectory (linear mixed-effects model).

Dental care utilization trajectory	*β* per 2-year wave (95% CI)	Change over 14 years (95% CI)	*p*
Slope, persistent high (reference)	−0.3272 (−0.3419, −0.3125)	−2.29 (−2.39, −2.19)	<0.0001
Additional decline vs. reference			
Decreasing	−0.0853 (−0.1181, −0.0525)	−0.60 (−0.83, −0.37)	<0.0001
Persistent low	−0.1070 (−0.1329, −0.0811)	−0.75 (−0.93, −0.57)	<0.0001
Contrast: persistent low vs. decreasing	−0.0217 (−0.0580, 0.0146)	−0.15 (−0.41, 0.10)	0.2410

Persistent High served as the reference group. Model adjusted for baseline age, sex, education, and race/ethnicity. CI, confidence interval.

**Figure 2:**
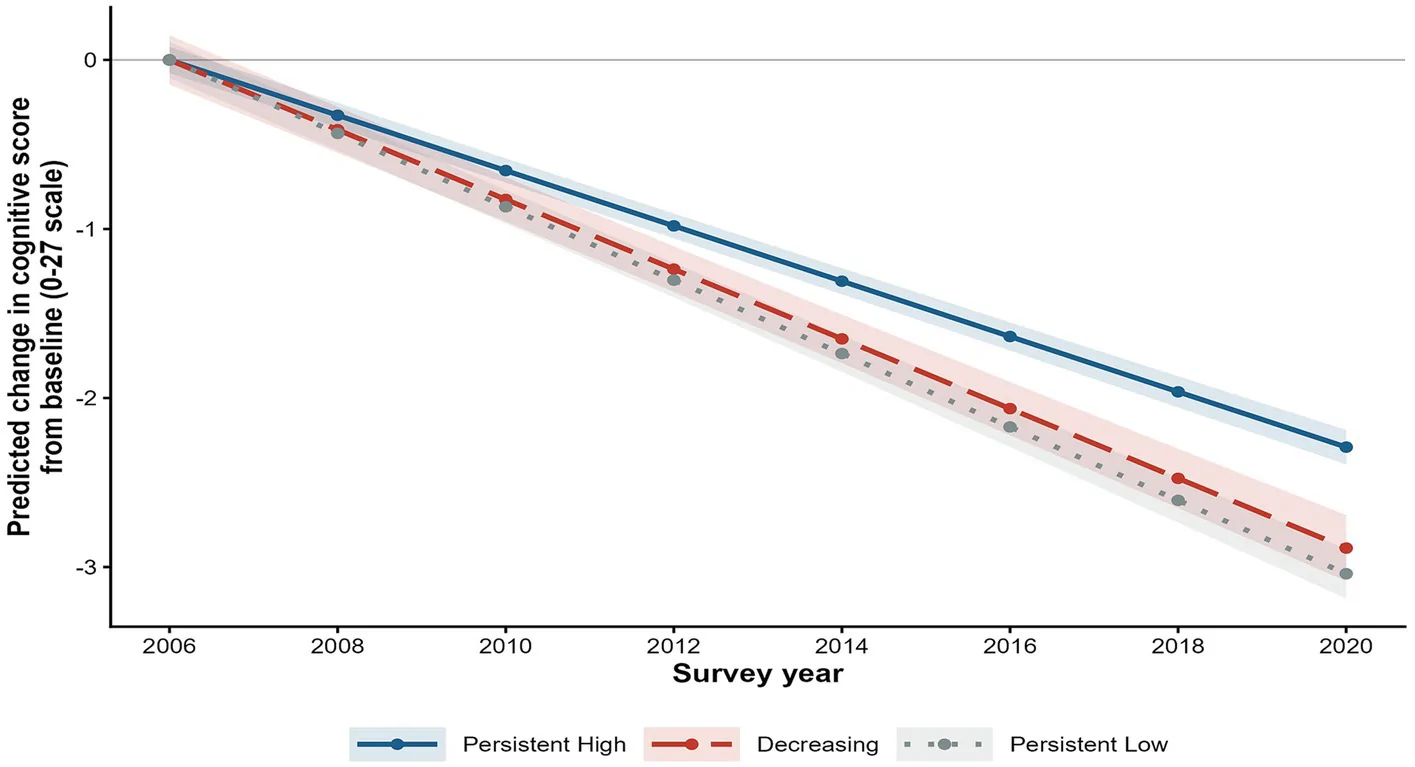
Predicted change in cognitive function from baseline according to dental care utilization trajectory. Predicted cognitive trajectories were estimated from the primary linear mixed-effects model. The structural mediation model reported in Table 3 used empirical Bayes slope estimates; therefore, coefficient magnitudes are not directly comparable between the two analyses.

The same pattern was obtained in the structural model used for the mediation analysis, in which the direct effects were −0.0463 (95% CI − 0.0578 to −0.0349) for the Decreasing group and −0.0465 (95% CI − 0.0550 to −0.0382) for the Persistent Low group, again with no statistically significant difference between them (*β* difference = −0.0002, 95% CI − 0.0121 to 0.0116, *p* = 0.965; Table 3, Supplementary Figure S2). These coefficients are smaller in magnitude than the mixed-model estimates because empirical Bayes slope estimates are shrunk toward the mean.

**Table 3:** Structural path coefficients from the serial mediation model (*n* = 14,576).

Path	*β*	95% CI	*p*
Trajectory → SRH slope			
Persistent low (a₂)	−0.0035	−0.0060, −0.0012	0.0036
Decreasing (a₃)	0.0019	−0.0011, 0.0049	0.2125
Trajectory → ADL slope			
Persistent low (e₂)	0.0392	0.0318, 0.0469	<0.0001
Decreasing (e₃)	0.0255	0.0166, 0.0345	<0.0001
Mediator → mediator			
SRH slope → ADL slope (b₁)	0.3660	0.3154, 0.4203	<0.0001
Mediators → outcome			
ADL slope → cognitive slope (c₁)	−0.1717	−0.1920, −0.1521	<0.0001
SRH slope → cognitive slope (d₁)	−0.1124	−0.1774, −0.0513	0.0005
Direct effects on cognitive slope			
Persistent low (c′₂)	−0.0465	−0.0550, −0.0382	<0.0001
Decreasing (c′₃)	−0.0463	−0.0578, −0.0349	<0.0001

Reference category: Persistent High. All models adjusted for baseline age, sex, education, and race/ethnicity. Coefficients are unstandardized; all slopes are expressed per biennial wave (2 years). The 95% confidence intervals were estimated from 5,000 bootstrap resamples. Twenty-three of the 14,599 analytic-sample participants were excluded from this model owing to missing baseline covariate data. CI, confidence interval; SRH, self-rated health; ADL, activities of daily living.

### 3.4 Serial mediation analysis

Both suboptimal trajectories showed significantly greater deterioration in ADL limitations than the Persistent High group (Decreasing: *β* = 0.0255, 95% CI 0.0166 to 0.0345; Persistent Low: *β* = 0.0392, 95% CI 0.0318 to 0.0469; both *p* < 0.0001; Table 3). For self-rated health, the Decreasing group did not differ significantly from the reference group (*β* = 0.0019, *p* = 0.213), whereas the Persistent Low group deteriorated more slowly (*β* = −0.0035, *p* = 0.0036).

A steeper increase in SRH scores (indicating worsening perceived health) was associated with greater deterioration in ADL limitations (*β* = 0.3660, 95% CI 0.3154 to 0.4203), and greater ADL deterioration was associated with faster cognitive decline (*β* = −0.1717, 95% CI − 0.1920 to −0.1521). The direct path from SRH slope to cognitive slope was also statistically significant (*β* = −0.1124, 95% CI − 0.1774 to −0.0513, *p* = 0.0005), although substantially smaller in magnitude than the ADL path.

Decomposition of the indirect effects (Table 4) showed that mediation operated almost entirely through the ADL pathway. The ADL-mediated indirect effects were significant for both groups (Decreasing: *β* = −0.0044; Persistent Low: *β* = −0.0067; both *p* < 0.0001), whereas the SRH-only indirect effect was small and, for the Persistent Low group, of the opposite sign (*β* = +0.0004, *p* = 0.029), reflecting the slower deterioration in perceived health in that group. The serial SRH → ADL pathway was negligible in magnitude (|*β*| ≤ 0.0002).

**Table 4:** Indirect effects and proportion mediated (*n* = 14,576).

Pathway	*β*	95% CI	*p*	Proportion mediated, % (95% CI)
Persistent low				
SRH-only indirect (a₂ × d₁)	0.0004	0.0001, 0.0008	0.0293	
ADL-mediated indirect (e₂ × c₁)	−0.0067	−0.0083, −0.0053	<0.0001	
Serial indirect (a₂ × b₁ × c₁)	0.0002	0.0001, 0.0004	0.0056	
Total indirect	−0.0061	−0.0077, −0.0046	<0.0001	11.6 (8.6–15.2)
Decreasing				
SRH-only indirect (a₃ × d₁)	−0.0002	−0.0006, 0.0001	0.2552	
ADL-mediated indirect (e₃ × c₁)	−0.0044	−0.0060, −0.0028	<0.0001	
Serial indirect (a₃ × b₁ × c₁)	−0.0001	−0.0003, 0.0001	0.2190	
Total indirect	−0.0047	−0.0065, −0.0031	<0.0001	9.3 (5.8–13.6)

Reference category: Persistent High. Proportion mediated = total indirect effect/(direct effect + total indirect effect). Path labels correspond to those in Table 3. The 95% confidence intervals were estimated from 5,000 bootstrap resamples. CI, confidence interval; SRH, self-rated health; ADL, activities of daily living.

Total indirect effects were significant for both groups (Decreasing: *β* = −0.0047, 95% CI − 0.0065 to −0.0031; Persistent Low: *β* = −0.0061, 95% CI − 0.0077 to −0.0046; both *p* < 0.0001) but accounted for only a modest share of the total association: 9.3% (95% CI 5.8–13.6) for the Decreasing group and 11.6% (95% CI 8.6–15.2) for the Persistent Low group. Because the direct associations remained substantially larger and significant, these findings indicate partial mediation, with most of the association unexplained by the measured pathways.

### 3.5 Sensitivity analyses

Findings were consistent across all sensitivity analyses (Supplementary Tables S3–S13). Direct associations, the ADL → cognition path, and total indirect effects retained their direction and statistical significance under survey weighting, additional adjustment for baseline cognition, depressive symptoms, household income and wealth, and after excluding participants with low baseline cognition. In the fully adjusted model, the proportion mediated attenuated to 6.6% (Decreasing) and 7.2% (Persistent Low) but remained significant. Additional adjustment for baseline smoking, alcohol consumption, and marital status did not alter the findings: all direct and indirect effects retained their direction and significance, with the direct effects modestly attenuated (Decreasing −0.0415; Persistent Low −0.0417; both *p* < 0.0001; Supplementary Table S9). To address reverse causation, we progressively excluded participants with prevalent or incident cognitive impairment (Supplementary Tables S10–S12). After excluding baseline dementia, CIND, and stroke (*n* = 10,896), and after additionally excluding those developing dementia or CIND within approximately 2 years (*n* = 9,838) and 4 years (*n* = 8,877), the direct associations and the ADL → cognition pathway remained negative and statistically significant throughout. Notably, the ADL → cognition coefficient did not attenuate across these exclusions and, if anything, increased in magnitude (−0.1717 in the main model; −0.2103, −0.2238, and −0.2039 in the three successive exclusions). Finally, when the baseline ADL limitation count was modeled as a covariate rather than longitudinal change in ADL limitations as a mediator, both suboptimal trajectories remained significantly associated with faster cognitive decline, with estimates essentially unchanged from the primary model (Supplementary Table S13).

In the lagged analysis (Supplementary Table S8), in which trajectory membership and mediator slopes were estimated over 2006–2016 and the cognitive slope over 2016–2020, the direct associations remained significant though attenuated (Decreasing: *β* = −0.0318, 95% CI − 0.0484 to −0.0171; Persistent Low: *β* = −0.0397, 95% CI − 0.0519 to −0.0283; both *p* < 0.0001), as did the ADL → cognition path (*β* = −0.0943, *p* < 0.0001) and the total indirect effects (proportion mediated 10.0 and 8.1%). In contrast, the direct path from SRH slope to cognitive slope was null in the lagged model (*β* = 0.0126, 95% CI − 0.0583 to 0.0808, *p* = 0.723).

A further exploratory model restricted to the first four waves (2006–2012) identified only two trajectory classes, persistently high and persistently low; the Decreasing trajectory emerged only when the full eight-wave observation period was modeled.

## 4 Discussion

In this nationally representative cohort of U.S. adults aged ≥50 years, we identified three long-term trajectories of dental care utilization. Both persistently low and declining utilization were associated with similarly faster cognitive decline than persistently high utilization, with no statistical evidence of a difference between the two estimates (*β* difference = −0.0217, 95% CI − 0.0580 to 0.0146, *p* = 0.241). Deterioration in physical functioning, indexed by worsening ADL limitations, accounted for the mediated portion of these associations, although mediation was modest (approximately 9–12%), indicating that most of the observed association was not explained by the measured pathways.

### 4.1 Two suboptimal trajectories, one common signal

An important feature of our findings is that the Persistent Low and Decreasing groups showed similar estimated rates of cognitive decline, with a formal contrast providing no evidence of a difference, despite markedly different baseline profiles: the Decreasing group entered follow-up with better education, better perceived health, and higher cognition than the Persistent Low group, yet declined at a comparable rate. These findings suggest that both persistently low utilization and a sustained decline from an initially high level may provide information not captured by dental attendance measured at a single time point. A person with historically high dental care utilization whose attendance subsequently declines may carry a signal of comparable magnitude to someone whose utilization remains persistently low. From a public health standpoint, this argues against a purely cross-sectional screening logic and in favor of monitoring change over time.

### 4.2 Functional deterioration remained the more robust pathway under temporal separation

In the primary (concurrent) mediation model, both the ADL and the SRH paths to cognitive decline were statistically significant. However, the two pathways diverged once the outcome window was separated from the exposure and mediator windows. In the lagged model, the ADL → cognition path remained robust, whereas the SRH → cognition path was null. This divergence suggests that the apparent association between perceived health and cognition reflects contemporaneous covariation or reverse-directional processes rather than a pathway in which deteriorating perceived health precedes cognitive decline. The ADL pathway, by contrast, remained significant when the outcome was measured in a subsequent, non-overlapping period.

We note that the lagged design separates the outcome from the exposure and mediator windows but does not fully separate the exposure from the mediators, which were estimated over the same 2006–2016 period. The lagged analysis therefore strengthens, but does not establish, the temporal ordering implied by the mediation model.

This pattern is consistent with a body of evidence linking functional decline and frailty with adverse cognitive aging (21, 22), and it suggests that long-term dental care utilization patterns primarily index broader functional vulnerability rather than subjective health appraisal. It also illustrates the interpretive risk of mediation models estimated entirely within a single concurrent observation window—a limitation that applies to much of the trajectory-mediation literature.

Notably, the Persistent Low group showed slower worsening of self-rated health than the reference group despite greater worsening of ADL limitations. This divergence is consistent with response shift, whereby individuals recalibrate their internal standards and report relatively stable health despite objective functional decline (30). It may also reflect poorer baseline self-rated health in this group, leaving less room for further measurable deterioration. The resulting small positive SRH-only indirect effect should not be read as a protective effect of poorer perceived health; it minimally offsets the substantially larger adverse pathway operating through functional deterioration.

### 4.3 Why long observation windows matter

In our data, the Decreasing trajectory was not identifiable when the observation period was restricted to the first four waves, suggesting that shorter follow-up may fail to capture gradual erosion in dental care utilization. This has a direct methodological implication: studies based on single-time-point measures or relatively short repeated-measure windows may therefore miss subgroups whose preventive engagement declines gradually over longer periods.

### 4.4 Interpretation and mechanisms

Beyond the inflammatory and masticatory mechanisms discussed above (7–9), declining dental care utilization may reflect reduced engagement with preventive health care and broader health-seeking behaviors accompanying functional deterioration (14–16). Dental care utilization is therefore best interpreted not solely as an indicator of oral health but as a behavioral marker reflecting multiple dimensions of health and healthy aging. Because clinical oral health measures were unavailable, our findings should not be interpreted as evidence that oral disease itself caused cognitive decline.

Reverse causation remains an important alternative explanation. Declining dental attendance may be an early behavioral manifestation of emerging cognitive impairment rather than a determinant of it, because sustaining regular attendance requires planning, transportation, financial resources, and continued engagement with preventive care. Adjustment for baseline cognition and the lagged analysis, in which the cognitive outcome was measured entirely after the exposure window (Section 4.2; Supplementary Table S8), both attenuate this concern. We examined it further by progressively excluding participants with prevalent and incident cognitive impairment (Supplementary Tables S10–S12). If the observed associations were largely driven by preclinical dementia affecting both dental attendance and subsequent cognition, excluding such individuals would be expected to attenuate the associations. Instead, the direct associations persisted and the ADL-mediated pathway did not weaken as prevalent and early incident cases were removed. This pattern reduces, although it does not eliminate, the likelihood that the findings are explained primarily by reverse causation from preclinical cognitive decline.

### 4.5 Magnitude and public health interpretation

The associations were small in absolute terms, corresponding to roughly 0.6–0.8 points of additional decline over 14 years on a 0–27 scale, against a background decline of approximately 2.3 points in the reference group. These modest differences are unlikely to support individual-level risk classification based on dental attendance alone. Their potential value may lie at the population level, particularly in settings where longitudinal dental attendance can be obtained from administrative or claims data. Consistent with our findings, a recent population-based analysis reported that regular dental visits were associated with a lower prevalence of subjective cognitive decline, independent of tooth loss and dementia risk factors (31). A sustained fall in routine dental attendance may serve as an inexpensive, passively collected signal identifying older adults who could benefit from closer functional and cognitive assessment. This implication should be regarded as hypothesis-generating.

### 4.6 Strengths and limitations

Strengths include a large, nationally representative cohort with 14 years of follow-up across eight biennial waves; the use of a cognitive measure administered consistently to self-respondents of all ages and in all waves; the integration of trajectory modeling with serial mediation analysis; a formal contrast between the two suboptimal trajectories; and a lagged design that separates the outcome window from the exposure and mediator windows.

Several limitations should be considered. First, the observational design precludes causal inference. In the primary mediation model, exposure trajectories, mediator slopes, and outcome slopes were derived from overlapping observation periods; the mediation analysis should therefore be read as describing longitudinal associations rather than causal pathways. The lagged analysis mitigates but does not eliminate this concern. Because individual slopes were estimated before entering the structural model, uncertainty associated with slope estimation may not have been fully propagated, and empirical Bayes shrinkage attenuated the path coefficients; direct mixed-model estimates of the same associations were correspondingly larger (Table 2, Figure 2). Furthermore, although the 27-point HRS summary score is validated for longitudinal population-level research, it does not comprehensively assess all cognitive domains and cannot substitute for clinical, neuropsychological, or biomarker-based diagnostic evaluation.

Second, dental care utilization was assessed with a single self-reported item that did not distinguish preventive from treatment-related visits, and no clinical oral health indicators (periodontal disease, tooth loss, denture use, chewing ability) were available. This heterogeneity may attenuate contrasts between trajectory groups, and the observed similarity between the Decreasing and Persistent Low groups could partly reflect the inability to separate preventive from disease-driven visits rather than true equivalence. This limitation reinforces our interpretation of dental care utilization as a broad behavioral marker rather than a specific clinical indicator of oral health.

Third, attrition due to death and loss to follow-up was substantial over 14 years, and 3,392 of 17,991 age-eligible 2006 respondents (18.9%) were excluded for having dental data in fewer than three waves. Excluded participants were markedly older and had poorer baseline cognition, self-rated health, and functional status (Supplementary Table S2). Because these characteristics are associated with both lower dental care utilization and faster cognitive decline, such selection may have affected the magnitude of the observed associations, although the direction of selection bias cannot be established with certainty.

Fourth, residual confounding by unmeasured factors—including dental insurance, health literacy, geographic access to dental care, and broader health-seeking behaviors—remains possible despite adjustment for major sociodemographic characteristics and, in sensitivity analyses, for income and wealth (14–16). Additional adjustment for smoking, alcohol consumption, and marital status did not materially change the estimates (Supplementary Table S9), and the downstream effects of these behaviors are in part captured by self-rated health and the ADL slope. We were unable to identify substance abuse reliably in these data, which remains an unmeasured potential confounder. In particular, because traditional Medicare does not cover routine dental care, dental attendance in this age group is strongly conditioned on income and supplemental dental insurance, which we were able to adjust for only partially.

Fifth, dental care utilization was modeled using a Gaussian latent-class specification on the probability scale rather than a logistic link, with linear class-specific slopes. Although predicted probabilities may extend beyond the 0–1 range and observed proportions in the Decreasing group deviated modestly from the fitted line at mid-follow-up, this specification was used solely for trajectory identification and yielded good classification quality (entropy = 0.881).

Sixth, the primary analyses did not fully account for the complex HRS sampling design. Survey weight–adjusted sensitivity analyses produced materially similar findings, but clustering and stratification were not modeled and may have affected variance estimation. Finally, our findings derive from a high-income setting. Access to and documentation of dental care differ markedly in low- and middle-income countries, where the majority of the global burden of oral disease is concentrated, the oral health workforce is scarce, and high out-of-pocket costs together with incomplete record-keeping constrain both the use and the longitudinal measurement of dental services (32). In such settings, dental care utilization may reflect barriers to access rather than health behavior, and its value as a behavioral marker may differ. Individuals who lack access to dental services, are economically disadvantaged, or have comorbidities severe enough to preclude dental care may also be underrepresented in this community-dwelling cohort. Although we adjusted for income and wealth in sensitivity analyses (Supplementary Tables S10–S12), residual differences in access and affordability may remain, and the generalizability of our findings beyond high-income contexts is therefore uncertain.

## 5 Conclusion

Long-term trajectories of dental care utilization were associated with cognitive aging among community-dwelling older adults. Persistently low and declining utilization were each associated with faster cognitive decline of a similar magnitude relative to persistently high utilization, with no statistical evidence of a difference between the two estimates. Deterioration in activities of daily living represented the principal mediating pathway, including when the outcome window was temporally separated from the exposure and mediator windows. Although the associations were small at the individual level, long-term patterns of dental care utilization may serve as accessible behavioral markers of broader functional vulnerability. Future studies incorporating objective clinical oral health measures are needed to clarify the mechanisms underlying these associations.

## Data Availability

Publicly available datasets were analyzed in this study. These data are available from the Health and Retirement Study (HRS), produced by the University of Michigan, at https://hrs.isr.umich.edu/data-products. The analysis used the Public Release Data (1992–2022 waves) and the RAND HRS Longitudinal File. No specific accession number is required, but registration and login are necessary to download the datasets.

## References

[1] Asher S Stephen R Mäntylä P Suominen AL Solomon A. Periodontal health, cognitive decline, and dementia: a systematic review and meta-analysis of longitudinal studies. J Am Geriatr Soc. (2022) 70:2695–709. doi: 10.1111/jgs.17978, 36073186 PMC9826143

[2] Qi X Zhu Z Plassman BL Wu B. Dose-response meta-analysis on tooth loss with the risk of cognitive impairment and dementia. J Am Med Dir Assoc. (2021) 22:2039–45. doi: 10.1016/j.jamda.2021.05.009, 34579934 PMC8479246

[3] Fang WL Jiang MJ Gu BB Wei YM Fan SN Liao W . Tooth loss as a risk factor for dementia: systematic review and meta-analysis of 21 observational studies. BMC Psychiatry. (2018) 18:345. doi: 10.1186/s12888-018-1927-0, 30342524 PMC6195976

[4] Tsakos G Watt RG Rouxel PL de Oliveira C Demakakos P. Tooth loss associated with physical and cognitive decline in older adults. J Am Geriatr Soc. (2015) 63:91–9. doi: 10.1111/jgs.13190, 25523131

[5] Choi M Park H Park B. Association of oral health and chewing ability with cognitive impairment and dementia in older adults: longitudinal findings from a national panel study. Arch Gerontol Geriatr. (2025) 138:105970. doi: 10.1016/j.archger.2025.105970, 40729797

[6] Qi X Zhu Z Wang K Zheng Y Li A Wu B. Association of gum treatment with cognitive decline and dementia risk among older adults with periodontal symptoms: a 12-year prospective cohort study. Neuroepidemiology. (2025) 59:313–22. doi: 10.1159/000540086, 39053434 PMC11759719

[7] Wang RP Ho YS Leung WK Goto T Chang RC. Systemic inflammation linking chronic periodontitis to cognitive decline. Brain Behav Immun. (2019) 81:63–73. doi: 10.1016/j.bbi.2019.07.002, 31279681

[8] Dominy SS Lynch C Ermini F Benedyk M Marczyk A Konradi A . *Porphyromonas gingivalis* in Alzheimer’s disease brains: evidence for disease causation and treatment with small-molecule inhibitors. Sci Adv. (2019) 5:eaau3333. doi: 10.1126/sciadv.aau3333, 30746447 PMC6357742

[9] Sta Maria MT Hasegawa Y Khaing AMM Salazar S Ono T. The relationships between mastication and cognitive function: a systematic review and meta-analysis. Jpn Dent Sci Rev. (2023) 59:375–88. doi: 10.1016/j.jdsr.2023.10.001, 38022390 PMC10630119

[10] Tanaka T Hirano H Ikebe K Ueda T Iwasaki M Minakuchi S . Consensus statement on "oral frailty" from the Japan geriatrics society, the Japanese Society of Gerodontology, and the Japanese association on sarcopenia and frailty. Geriatr Gerontol Int. (2024) 24:1111–9. doi: 10.1111/ggi.14980, 39375858 PMC11843523

[11] Nagatani M Tanaka T Son BK Kawamura J Tagomori J Hirano H . Oral frailty as a risk factor for mild cognitive impairment in community-dwelling older adults: Kashiwa study. Exp Gerontol. (2023) 172:112075. doi: 10.1016/j.exger.2022.112075, 36581224

[12] Tanaka T Lyu W Hirano H Shirobe M Iijima K. Oral frailty and the trajectories of psychological well-being and cognitive function: findings from the 12-year community-based Kashiwa study. J Gerontol A Biol Sci Med Sci. (2026) 81:glag006. doi: 10.1093/gerona/glag006, 41533683

[13] Do KY Won CW Kim M Lim JY. Oral functional limitation and risk of frailty onset in older adults: a sex-stratified 4-year cohort study with competing risk analysis. GeroScience. (2025). doi: 10.1007/s11357-025-02015-8, 41331404 PMC13601412

[14] Kailembo A Quiñonez C Lopez Mitnik GV Weintraub JA Stewart Williams J Preet R . Income and wealth as correlates of socioeconomic disparity in dentist visits among adults aged 20 years and over in the United States, 2011–2014. BMC Oral Health. (2018) 18:147. doi: 10.1186/s12903-018-0613-4, 30139349 PMC6108097

[15] Manski RJ Schimmel Hyde J Chen H Moeller JF. Differences among older adults in the types of dental services used in the United States. Inquiry. (2016) 53:0046958016652523. doi: 10.1177/0046958016652523, 27284127 PMC4905592

[16] Cademartori MG Gastal MT Nascimento GG Demarco FF Corrêa MB. Is depression associated with oral health outcomes in adults and elders? A systematic review and meta-analysis. Clin Oral Investig. (2018) 22:2685–702. doi: 10.1007/s00784-018-2611-y, 30191327

[17] Nagin DS. Group-Based Modeling of Development. Cambridge, MA: Harvard University Press (2005).

[18] Nagin DS Odgers CL. Group-based trajectory modeling in clinical research. Annu Rev Clin Psychol. (2010) 6:109–38. doi: 10.1146/annurev.clinpsy.121208.131413, 20192788

[19] Kaur G King T Karahalios A Singh A. Longitudinal trajectories of dental attendance in Australian adults. J Dent Res. (2025) 104:604–10. doi: 10.1177/00220345251315155, 40070224 PMC12075884

[20] Testa A Mijares L Santos MR Jackson DB Semenza DC. Dental care trajectories among formerly incarcerated older adults in the United States. PLoS One. (2025) 20:e0320658. doi: 10.1371/journal.pone.0320658 PMC11978076 40198718

[21] Li R Liu Z Huang R Ma Y Zhao Y Chen S . Frailty trajectory predicts subsequent cognitive decline: a 26-year population-based longitudinal cohort study. MedComm. (2023) 4:e296. doi: 10.1002/mco2.296 PMC10242271 37287754

[22] Guo CY Sun Z Tan CC Tan L Xu W. Multi-concept frailty predicts the late-life occurrence of cognitive decline or dementia: an updated systematic review and meta-analysis of longitudinal studies. Front Aging Neurosci. (2022) 14:855553. doi: 10.3389/fnagi.2022.855553, 35645771 PMC9131093

[23] Idler EL Benyamini Y. Self-rated health and mortality: a review of twenty-seven community studies. J Health Soc Behav. (1997) 38:21–37. doi: 10.2307/2955359, 9097506

[24] Sonnega A Faul JD Ofstedal MB Langa KM Phillips JWR Weir DR. Cohort profile: the health and retirement study (HRS). Int J Epidemiol. (2014) 43:576–85. doi: 10.1093/ije/dyu067, 24671021 PMC3997380

[25] Ofstedal MB Fisher GG Herzog AR. Documentation of Cognitive Functioning Measures in the Health and Retirement Study . HRS Documentation Report DR-006. Ann Arbor, MI: University of Michigan (2005).

[26] Turvey CL Wallace RB Herzog R. A revised CES-D measure of depressive symptoms and a DSM-based measure of major depressive episodes in the elderly. Int Psychogeriatr. (1999) 11:139–48. doi: 10.1017/S1041610299005694, 11475428

[27] Proust-Lima C Philipps V Liquet B. Estimation of extended mixed models using latent classes and latent processes: the R package lcmm. J Stat Softw. (2017) 78:1–56. doi: 10.18637/jss.v078.i02

[28] Rosseel Y. Lavaan: an R package for structural equation modeling. J Stat Softw. (2012) 48:1–36. doi: 10.18637/jss.v048.i02

[29] Preacher KJ Hayes AF. Asymptotic and resampling strategies for assessing and comparing indirect effects in multiple mediator models. Behav Res Methods. (2008) 40:879–91. doi: 10.3758/BRM.40.3.879, 18697684

[30] Sprangers MA Schwartz CE. Integrating response shift into health-related quality of life research: a theoretical model. Soc Sci Med. (1999) 48:1507–15. doi: 10.1016/S0277-9536(99)00045-3, 10400253

[31] Alshanbari MH Cheney AM Alhazmi HA Bouldin ED. The impact of oral health and dental services on the prevalence of subjective cognitive decline among middle-aged and older US adults: behavioral risk factor surveillance system, 2022. Prev Chronic Dis. (2025) 22:250083. doi: 10.5888/pcd22.250083, 40907543 PMC12416401

[32] World Health Organization. Global Oral Health Status Report: Towards Universal Health Coverage for Oral Health by 2030. Geneva: World Health Organization (2022).

