# Supplementary material for: Long-term dental care utilization trajectories as behavioral markers of cognitive aging: a 14-year national cohort study
Source: Front Public Health. 2026 Sep 24;14:1938854. doi: 10.3389/fpubh.2026.1938854 (PMC13645594; doi:10.3389/fpubh.2026.1938854)
Supplement: Supplementary file 1 [file Supplementary_File_1.docx]

**Supplementary Figure S1.** **Flowchart of participant selection.** Exclusion for fewer than three waves of dental care utilization data was predominantly attributable to death or loss to follow-up over the 14-year observation period. HRS, Health and Retirement Study.

**Supplementary Figure S2. Direct associations between dental care utilization trajectory and the rate of cognitive decline. Points represent unstandardized coefficients (additional change in cognitive score per biennial wave); the 95% confidence intervals were estimated from 5,000 bootstrap resamples. Persistent High served as the reference. Adjusted for baseline age, sex, education, and race/ethnicity.**

**Supplementary Figure S3. Serial mediation pathways linking dental care utilization trajectories to cognitive decline. Values are unstandardized coefficients from the adjusted serial mediation model (n = 14,576). Where two coefficients are shown, they correspond to the Persistent Low and Decreasing trajectories, respectively; Persistent High served as the reference. The Decreasing → SRH slope path was not significant; all other displayed paths were significant. *P < 0.05, **P < 0.01, ***P < 0.001. SRH, self-rated health; ADL, activities of daily living.**

Supplementary Table S1. Fit statistics for group-based trajectory models with two to six classes

| Classes | BIC | Entropy | Empty classes | Smallest non-empty class, % | APP range | Converged |
| --- | --- | --- | --- | --- | --- | --- |
| 2 | 81,116.2 | 0.935 | 0 | 40.0 | 0.978–0.989 | Yes |
| **3 (selected)** | **75,034.1** | **0.881** | **0** | **14.5** | **0.833–0.967** | **Yes** |
| 4 | 75,062.9 | 0.640 | 1 | 15.8 | 0.491–0.967 | Yes |
| 5 | 75,091.7 | 0.557 | 2 | 16.1 | 0.359–0.967 | Yes |
| 6 | 71,001.1 | 0.596 | 2 | 9.0 | 0.376–0.933 | Yes |

The three-class solution was selected because it provided the best overall balance of model fit, classification quality, class size, and substantive interpretability. Compared with the two-class solution, it had a substantially lower BIC and identified three distinct and interpretable trajectories, with no empty classes and adequate entropy and average posterior probabilities. Models with four or more classes produced one or more empty classes and substantially poorer classification quality. Although the six-class solution had a lower BIC, its two empty classes and poor classification quality indicated over-extraction. A quadratic specification of time was also examined for the three-class solution; it yielded a modestly lower BIC (74,800.4) but nearly identical class membership (97.5% agreement with the linear solution) and the same substantive ordering of the trajectories. The more parsimonious linear specification was therefore retained. BIC, Bayesian Information Criterion; APP, average posterior probability of class assignment.

Supplementary Table S2. Comparison of included and excluded age-eligible 2006 respondents

| Characteristic | Included  (n = 14,599) | Excluded  (n = 3,392) | SMD |
| --- | --- | --- | --- |
| Age, years, mean (SD) | 67.45 (9.64) | 76.09 (11.51) | 0.813 |
| Female, n (%) | 8,627 (59.1) | 1,885 (55.6) | 0.071 |
| Education, years, mean (SD) | 12.54 (3.22) | 11.64 (3.49) | 0.270 |
| Race/ethnicity, n (%) |  |  | 0.058 |
| — White | 10,884 (74.6) | 2,567 (75.7) |  |
| — Black | 2,011 (13.8) | 489 (14.4) |  |
| — Other | 1,703 (11.7) | 336 (9.9) |  |
| Depressive symptoms (CES-D, 0–8), median [IQR] | 1.00 [0.00, 2.00] | 1.00 [0.00, 3.00] | 0.242 |
| Self-rated health (1–5), mean (SD) | 2.77 (1.09) | 3.45 (1.15) | 0.611 |
| ADL limitations (0–5), median [IQR] | 0.00 [0.00, 0.00] | 0.00 [0.00, 2.00] | 0.623 |
| Cognitive score (0–27), mean (SD) | 15.55 (4.42) | 12.66 (5.13) | 0.603 |

Excluded participants were age-eligible respondents in 2006 with dental care utilization data available for fewer than three survey waves, primarily because of death or loss to follow-up. Their less favorable baseline health profiles suggest the possibility of selection bias. SMD, standardized mean difference; ADL, activities of daily living; CES-D, Center for Epidemiologic Studies Depression Scale.

Supplementary Tables S3–S7. Sensitivity analyses of the serial mediation model

| Parameter | Main model | S3: Survey weight–adjusted | S4: + baseline cognition | S5: + baseline cognition and CES-D | S6: fully adjusted† | S7: excluding low baseline cognition‡ |
| --- | --- | --- | --- | --- | --- | --- |
| SRH slope → ADL slope (b₁) | 0.3660 (0.3154, 0.4203) | 0.3418 (0.2886, 0.3949) | 0.4098 (0.3571, 0.4593) | 0.4581 (0.4077, 0.5090) | 0.4707 (0.4207, 0.5214) | 0.3953 (0.3425, 0.4486) |
| ADL slope → cognitive slope (c₁) | −0.1717 (−0.1920, −0.1521) | −0.1946 (−0.2183, −0.1709) | −0.1877 (−0.2078, −0.1662) | −0.1798 (−0.2009, −0.1600) | −0.1766 (−0.1980, −0.1563) | −0.2018 (−0.2258, −0.1763) |
| SRH slope → cognitive slope (d₁) | −0.1124 (−0.1774, −0.0513) | −0.1127 (−0.1866, −0.0389) | −0.1019 (−0.1638, −0.0423) | −0.1289 (−0.1995, −0.0633) | −0.1363 (−0.2058, −0.0691) | −0.1422 (−0.2154, −0.0680) |
| Direct effect, Persistent Low | −0.0465 (−0.0550, −0.0382) | −0.0510 (−0.0613, −0.0407) | −0.0504 (−0.0595, −0.0421) | −0.0487 (−0.0569, −0.0402) | −0.0444 (−0.0527, −0.0357) | −0.0466 (−0.0568, −0.0368) |
| Direct effect, Decreasing | −0.0463 (−0.0578, −0.0349) | −0.0444 (−0.0583, −0.0306) | −0.0490 (−0.0603, −0.0365) | −0.0475 (−0.0602, −0.0364) | −0.0451 (−0.0575, −0.0340) | −0.0489 (−0.0619, −0.0374) |
| Total indirect, Persistent Low | −0.0061 (−0.0077, −0.0046) | −0.0070 (−0.0088, −0.0052) | −0.0056 (−0.0072, −0.0040) | −0.0048 (−0.0064, −0.0034) | −0.0034 (−0.0050, −0.0019) | −0.0066 (−0.0084, −0.0049) |
| Total indirect, Decreasing | −0.0047 (−0.0065, −0.0031) | −0.0060 (−0.0080, −0.0041) | −0.0043 (−0.0062, −0.0025) | −0.0039 (−0.0057, −0.0022) | −0.0032 (−0.0050, −0.0015) | −0.0046 (−0.0067, −0.0026) |
| Proportion mediated, Persistent Low, % | 11.6 | 12.0 | 10.0 | 9.0 | 7.2 | 12.4 |
| Proportion mediated, Decreasing, % | 9.3 | 12.0 | 8.1 | 7.6 | 6.6 | 8.5 |

Values are unstandardized coefficients (95% confidence intervals). All models were adjusted for baseline age, sex, education, and race/ethnicity unless otherwise indicated. †Additionally adjusted for baseline cognition, depressive symptoms, log-transformed household income, and inverse hyperbolic sine–transformed household wealth. ‡Excluding participants with a baseline cognitive score of 10 or below. The primary model used 5,000 bootstrap resamples; sensitivity analyses used 1,000. CES-D, Center for Epidemiologic Studies Depression Scale; SRH, self-rated health; ADL, activities of daily living.

Supplementary Table S8. Lagged design sensitivity analysis

| Parameter | β | 95% CI | P |
| --- | --- | --- | --- |
| SRH slope → ADL slope (b₁) | 0.3751 | 0.3269, 0.4288 | <0.0001 |
| ADL slope → cognitive slope (c₁) | −0.0943 | −0.1314, −0.0537 | <0.0001 |
| **SRH slope → cognitive slope (d₁)** | **0.0126** | **−0.0583, 0.0808** | **0.7229** |
| Direct effect, Persistent Low | −0.0397 | −0.0519, −0.0283 | <0.0001 |
| Direct effect, Decreasing | −0.0318 | −0.0484, −0.0171 | <0.0001 |
| Total indirect, Persistent Low | −0.0035 | −0.0052, −0.0020 | <0.0001 |
| Total indirect, Decreasing | −0.0036 | −0.0055, −0.0019 | <0.0001 |
| Proportion mediated, Persistent Low, % | 8.1 | 4.4, 13.4 | 0.0004 |
| Proportion mediated, Decreasing, % | 10.0 | 5.0, 19.7 | 0.0083 |

Trajectory membership and mediator slopes were estimated over 2006–2016 (waves 8–13), and the cognitive slope over 2016–2020 (waves 13–15), thereby separating the outcome window from the exposure and mediator windows. Trajectory classes were re-estimated within the 2006–2016 window (Persistent High, *n* = 8,015; Persistent Low, *n* = 4,827; Decreasing, *n* = 1,757). Models were adjusted for baseline age, sex, education, and race/ethnicity, with 1,000 bootstrap resamples. CI, confidence interval; SRH, self-rated health; ADL, activities of daily living.

**Supplementary Table S9. Serial mediation model additionally adjusted for smoking, alcohol consumption, and marital status**

The fully specified serial mediation model was additionally adjusted for baseline current smoking, any alcohol consumption, and marital/partnership status (n = 13,850; 749 of the 14,599 analytic-sample participants were excluded for missing data on the additional covariates).

| Parameter | β | 95% CI | P |
| --- | --- | --- | --- |
| SRH slope → ADL slope (b₁) | 0.4700 | 0.4198, 0.5271 | <0.0001 |
| ADL slope → cognitive slope (c₁) | −0.1767 | −0.1955, −0.1562 | <0.0001 |
| SRH slope → cognitive slope (d₁) | −0.1335 | −0.2019, −0.0637 | 0.0001 |
| Direct effect, Decreasing | −0.0415 | −0.0530, −0.0301 | <0.0001 |
| Direct effect, Persistent Low | −0.0417 | −0.0514, −0.0317 | <0.0001 |
| Total indirect, Decreasing | −0.0040 | −0.0057, −0.0023 | <0.0001 |
| Total indirect, Persistent Low | −0.0037 | −0.0053, −0.0021 | <0.0001 |
| Proportion mediated, Decreasing, % | 8.8 | 5.1, 13.6 | <0.0001 |
| Proportion mediated, Persistent Low, % | 8.1 | 4.7, 12.1 | <0.0001 |

All coefficients are unstandardized; reference category is Persistent High. Adjusted for baseline age, sex, education, race/ethnicity, baseline cognition, depressive symptoms, log household income, inverse-hyperbolic-sine–transformed household wealth, current smoking, any alcohol consumption, and marital/partnership status. The 95% confidence intervals were estimated from 1,000 bootstrap resamples. Direct effects were modestly attenuated relative to the main model (Table 3). CI, confidence interval; SRH, self-rated health; ADL, activities of daily living.

**Supplementary Table S10. Serial mediation model excluding participants with baseline dementia, CIND, or stroke**

Participants classified at baseline as having dementia (27-point score 0–6) or cognitive impairment no dementia (CIND; 7–11) according to the Crimmins et al. (2011) cut-points, or reporting a history of stroke, were excluded (n = 10,896).

| Parameter | β | 95% CI | P |
| --- | --- | --- | --- |
| ADL slope → cognitive slope (c₁) | −0.2103 | −0.2376, −0.1816 | <0.0001 |
| Direct effect, Decreasing | −0.0522 | −0.0646, −0.0399 | <0.0001 |
| Direct effect, Persistent Low | −0.0467 | −0.0579, −0.0362 | <0.0001 |
| Total indirect, Decreasing | −0.0050 | −0.0071, −0.0029 | <0.0001 |
| Total indirect, Persistent Low | −0.0048 | −0.0067, −0.0029 | <0.0001 |

Reference category is Persistent High. Adjusted for baseline age, sex, education, race/ethnicity, baseline cognition, depressive symptoms, log household income, and inverse-hyperbolic-sine–transformed household wealth. The 95% confidence intervals were estimated from 1,000 bootstrap resamples. The direct associations, total indirect effects, and the ADL-to-cognition association remained negative and statistically significant after excluding participants with prevalent cognitive impairment or stroke. CI, confidence interval; ADL, activities of daily living; CIND, cognitive impairment no dementia.

**Supplementary Table S11. Serial mediation model additionally excluding incident cognitive impairment within approximately two years**

In addition to the baseline exclusions in Supplementary Table S10, participants who developed dementia or CIND by wave 9 (~2 years after baseline) were excluded (n = 9,838).

| Parameter | β | 95% CI | P |
| --- | --- | --- | --- |
| ADL slope → cognitive slope (c₁) | −0.2238 | −0.2589, −0.1888 | <0.0001 |
| Direct effect, Decreasing | −0.0519 | −0.0663, −0.0382 | <0.0001 |
| Direct effect, Persistent Low | −0.0423 | −0.0537, −0.0308 | <0.0001 |
| Total indirect, Decreasing | −0.0037 | −0.0061, −0.0014 | 0.0012 |
| Total indirect, Persistent Low | −0.0043 | −0.0063, −0.0021 | <0.0001 |

Reference category is Persistent High. Adjustment (including household income and wealth) and bootstrap resampling as in Supplementary Table S10. CI, confidence interval; ADL, activities of daily living; CIND, cognitive impairment no dementia.

**Supplementary Table S12. Serial mediation model additionally excluding incident cognitive impairment within approximately four years**

As in Supplementary Table S11, but additionally excluding participants who developed dementia or CIND through wave 10 (~4 years after baseline) (n = 8,877).

| Parameter | β | 95% CI | P |
| --- | --- | --- | --- |
| ADL slope → cognitive slope (c₁) | −0.2039 | −0.2393, −0.1702 | <0.0001 |
| Direct effect, Decreasing | −0.0433 | −0.0575, −0.0290 | <0.0001 |
| Direct effect, Persistent Low | −0.0371 | −0.0488, −0.0252 | <0.0001 |
| Total indirect, Decreasing | −0.0040 | −0.0062, −0.0019 | 0.0004 |
| Total indirect, Persistent Low | −0.0043 | −0.0062, −0.0024 | <0.0001 |

Reference category is Persistent High. Adjustment (including household income and wealth) and bootstrap resampling as in Supplementary Table S10. Across the progressive exclusion analyses (Supplementary Tables S10–S12), the direct associations and total indirect effects remained negative and statistically significant, and the ADL-to-cognition coefficient did not attenuate. This pattern reduces, although it does not eliminate, the likelihood that the findings are explained primarily by reverse causation from preclinical cognitive decline. CI, confidence interval; ADL, activities of daily living; CIND, cognitive impairment no dementia.

**Supplementary Table S13. Adjusted associations between dental care utilization trajectory and cognitive decline, with baseline ADL modeled as a covariate rather than a mediator**

To address the possibility that functional status acts as a determinant of dental care utilization rather than as a mediator, the baseline ADL limitation count (range 0–5) was included as a covariate, and the associations of trajectory group with the rate of cognitive decline were re-estimated (n = 13,947).

| **Dental care utilization trajectory** | **β** | **95% CI** | **P** |
| --- | --- | --- | --- |
| Persistent High | Reference | — | — |
| Decreasing | −0.047 | −0.059, −0.037 | <0.0001 |
| Persistent Low | −0.049 | −0.059, −0.040 | <0.0001 |

Coefficients are unstandardized; the Persistent High trajectory was the reference category. The baseline ADL limitation count (range 0–5) was included as a covariate rather than modeling longitudinal change in ADL limitations as a mediator. The model corresponded to the fully adjusted model—baseline age, sex, education, race/ethnicity, baseline cognition, depressive symptoms, log household income, and inverse-hyperbolic-sine–transformed household wealth—with the baseline ADL limitation count additionally included as a covariate. The behavioral and social covariates examined separately in Supplementary Table S9 were not included. The 95% confidence intervals were estimated from 1,000 bootstrap resamples. The associations remained similar in magnitude to those in the primary analysis, supporting robustness to the alternative specification of baseline ADL as a covariate. ADL, activities of daily living; CI, confidence interval.
